# SPTBN1 suppresses the progression of epithelial ovarian cancer via SOCS3-mediated blockade of the JAK/STAT3 signaling pathway

**DOI:** 10.18632/aging.103303

**Published:** 2020-06-08

**Authors:** Mo Chen, Jia Zeng, Shuyi Chen, Jiajia Li, Huijie Wu, Xuhui Dong, Yuan Lei, Xiuling Zhi, Liangqing Yao

**Affiliations:** 1 Department of Gynecology, Obstetrics and Gynecology Hospital, Fudan University, Shanghai 200011, China; 2 Department of Physiology and Pathophysiology, School of Basic Medical Sciences, Fudan University, Shanghai 200032, China

**Keywords:** SPTBN1, epithelial ovarian cancer, SOCS3, JAK/STAT pathway, EMT

## Abstract

SPTBN1 plays an anticancer role in many kinds of tumors and participates in the chemotherapeutic resistance of epithelial ovarian cancer (EOC). Here, we reported that lower SPTBN1 expression was significantly related to advanced EOC stage and shorter progression-free survival. SPTBN1 expression was also higher in less invasive EOC cell lines. Moreover, SPTBN1 decreased the migration ability of the EOC cells A2780 and HO8910 and inhibited the growth of EOC cells in vitro and tumor xenografts in vivo. SPTBN1 suppression increased the epithelial mesenchymal transformation marker Vimentin while decreasing E-cadherin expression. By analyzing TCGA data and immunohistochemistry staining of tumor tissue, we found that SPTBN1 and SOCS3 were positively coexpressed in EOC patients. SOCS3 overexpression or JAK2 inhibition decreased the proliferation and migration of EOC cells as well as the expression of p-JAK2, p-STAT3 and Vimentin, which were enhanced by the downregulation of SPTBN1, while E-cadherin expression was also reversed. It was also verified in mouse embryonic fibroblasts (MEFs) that loss of SPTBN1 activated the JAK/STAT3 signaling pathway with suppression of SOCS3. Our results suggest that SPTBN1 suppresses the progression of epithelial ovarian cancer via SOCS3-mediated blockade of the JAK/STAT3 signaling pathway.

## INTRODUCTION

Epithelial ovarian cancer (EOC) is the third most common gynecological tumor with the highest mortality rate. Despite various treatments, including surgery, chemotherapy, radiotherapy, targeted therapy and immunotherapy, it is still likely for recurrence or metastasis to occur, with a 5-year survival rate of 35%-57% [1]. Therefore, there is an urgent need to understand the mechanisms of EOC progression to develop biomarkers and therapeutics for this disease.

SPTBN1 (spectrin beta chain, non-erythrocytic 1), also known as ELF, encodes the beta II subunit of spectrin (βIISP, a cytoskeleton protein) to maintain cell morphology [2] and is involved in regulating DNA damage repair [3, 4], angiogenesis [5] and stemness maintenance [6, 7]. Moreover, SPTBN1 is an adaptor protein for Smad3/Smad4 complex formation in the TGF-β signaling pathway, thereby participating in cell cycle regulation [8].

It has also been reported that SPTBN1 plays an important role in the pathogenesis and progression of many tumors, such as liver cancer [5–7, 9, 10], lung cancer [11–14], pancreatic cancer [15] and colon cancer [16, 17]. Regarding EOC, studies have shown that SPTBN1 induces chemotherapeutic resistance by forming the spectrin-GS-pt complex [18]. The expression of SPTBN1 in EOC patients with BRCA mutations is 2.5 times lower than that in patients with wild-type BRCA [19]. However, the effects of SPTBN1 on the growth and metastasis of epithelial ovarian cancer and the specific mechanism have rarely been reported and need to be further studied.

The SOCS (suppressors of cytokine signaling) family includes cytokine signal suppressors, which can negatively regulate the Janus kinase (JAK)/signal transducer and activator of transcription (STAT) signaling pathway [20, 21]. Deletion of the suppressor of cytokine signaling 3 (SOCS3) gene and activation of the JAK-STAT signaling pathway are closely related to hepatocarcinogenesis [22], gastric cancer [23], malignant fibrous histiocytoma [24], as well as the occurrence, development and metastasis of EOC [25–27]. Since cytokines such as interferon-γ (IFNγ) play an important role in antitumor immunity, the SOCS-JAK interaction has also been demonstrated as a potentially druggable target for cancer immunotherapy [28].

Epithelial mesenchymal transformation (EMT) is a biological process during development by which epithelial cells acquire characteristics of mesenchymal cells. Abnormal cell proliferation and EMT are also closely related to epithelial cancer tumorigenesis, progression and metastasis. As a key step in the early stage of cancer metastasis, EMT can be regulated by a variety of signaling pathways, including JAK/STAT3 and TGF-β/Smad [29]. However, the interaction between these two pathways in EOC EMT is still not clear, although suppression of SPTBN1 and SMAD3 promoted the transcription of STAT3 in hepatocellular carcinoma [30].

In this study, we investigated the role of SPTBN1 in the development and metastasis of epithelial ovarian cancer and its molecular mechanisms. Our work revealed that SPTBN1 suppresses the growth and metastasis of epithelial ovarian cancer via SOCS3-mediated blockade of the JAK/STAT3 signaling pathway.

## RESULTS

### The expression of SPTBN1 is closely related to the progression of epithelial ovarian cancer

**Figure 1:**
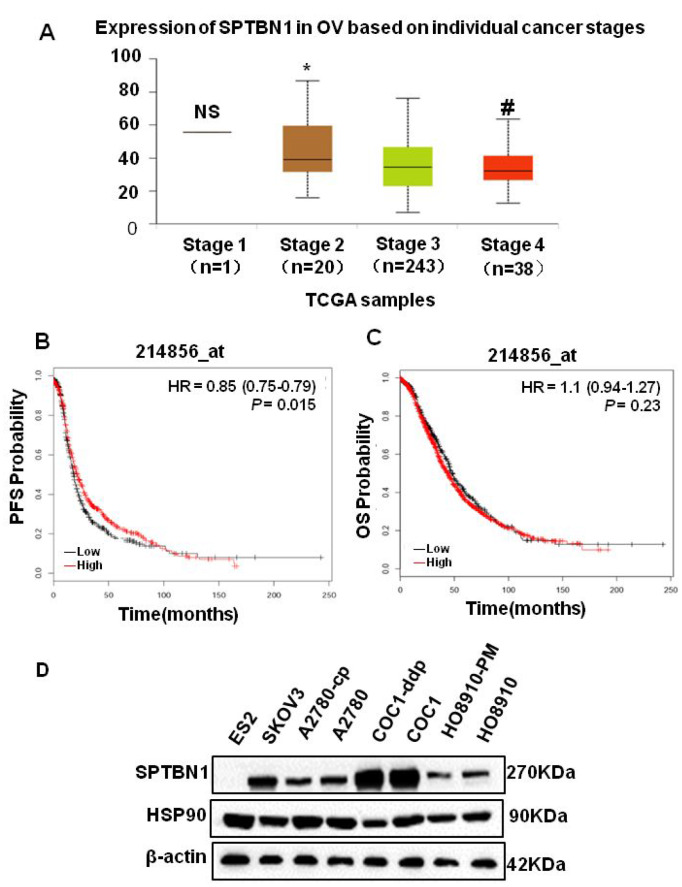
**SPTBN1 is closely related to the progression of epithelial ovarian cancer.** (**A**) Analysis of TCGA data. The expression of SPTBN1 was significantly decreased as EOC staging increased. NS: stage 1 vs stage 2, 3, and 4; *P<0.05 vs stage 3 and 4; #*P*<0.05 vs stage 3. (**B**, **C**) The progression-free survival (PFS) and overall survival (OS) rates were retrieved from the TCGA dataset (probe 214856_at) and compared between the low-SPTBN1 and high-SPTBN1 ovarian cancer patients. The low-SPTBN1 group had shorter PFS than the high-SPTBN1 group. (**D**) Expression of SPTBN1 in 8 ovarian cancer cell lines. The expression of SPTBN1 in high-metastasis HO8910-PM cells was lower than that in conventional HO8910 cells, suggesting that the decrease in SPTBN1 expression may be correlated with the increased incidence of malignancy of epithelial ovarian cancer.

By analyzing TCGA data, we found that the expression of SPTBN1 was significantly decreased as EOC staging increased (Figure 1A). The progression-free survival (PFS) was shorter in patients with low SPTBN1 expression (Figure 1B), but the SPTBN1 level did not affect the overall survival (OS) of patients with EOC (Figure 1C). Then, the expression of SPTBN1 in eight ovarian cancer cell lines was measured by western blotting. Compared with that in HO8910 cells, the expression of SPTBN1 in highly metastatic HO8910-PM cells was lower. In most malignant ES2 cells, no SPTBN1 was detected (Figure 1D). These results suggested that decreased levels of SPTBN1 were related to invasive progression and worse prognosis of epithelial ovarian cancer.

### SPTBN1 inhibits EOC cell growth in vitro and in vivo

**Figure 2:**
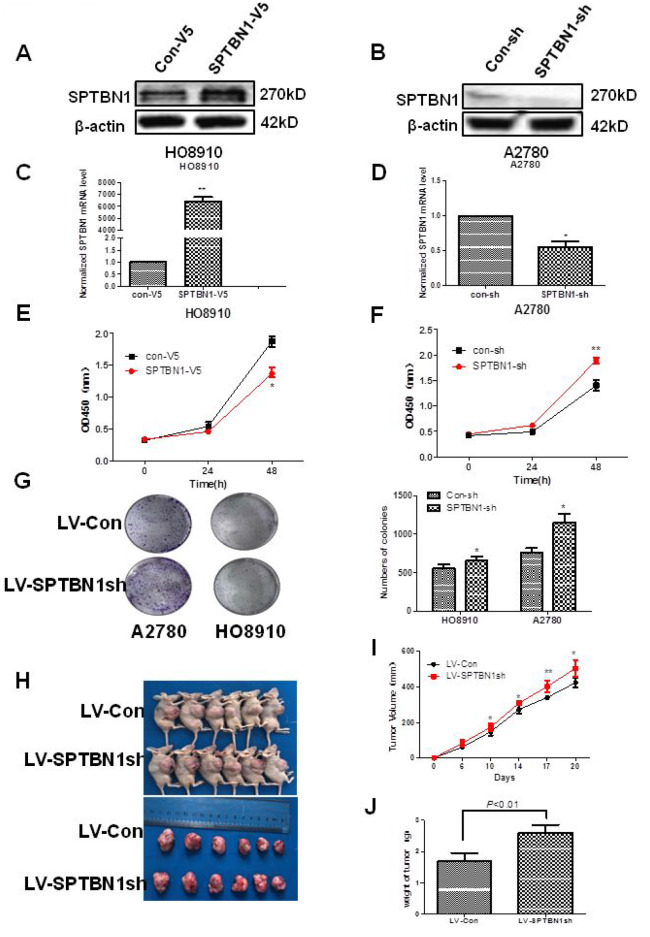
**SPTBN1 inhibits the growth of epithelial ovarian cancer cells in vitro and in vivo.** (**A**, **B**) The protein level of SPTBN1 was detected by western blot. (**C**, **D**) The mRNA level of SPTBN1 was analyzed by real-time PCR. SPTBN1 was overexpressed in HO8910 cells but decreased in A2780 cells after transfection with the SPTBN1-V5 or SPTBN1-sh plasmid, respectively. *P < 0.05 vs Con-V5, **P < 0.01 vs Con-sh, n=3. (**E**, **F**) Cell viability was evaluated by CCK8 assay. **P < 0.01 vs Con-V5, *P < 0.05 vs Con-sh, n=3. (**G**) Cell proliferation was assessed by colony formation assay. Loss of SPTBN1 promotes the proliferation of A2780 and HO8910 cells. *P < 0.05 vs Con-sh, n=3; (**H**–**J**) Mouse xenograft tumors derived from A2780 cells. Loss of SPTBN1 promotes the growth of epithelial ovarian cancer cells in vivo. (**H**) Images of mice with tumors (upper) and harvested tumors for each treatment group (lower). (**I**) Tumor growth curves. (**J**) Tumor weight at sacrifice. *P < 0.05; **P < 0.01 vs LV-Con.

As shown in Figure 2, SPTBN1 was overexpressed in SPTBN1-V5-transfected HO8910 cells (Figure 2A, 2C) and suppressed in SPTBN1sh-transfected A2780 cells (Figure 2B, 2D) both at the mRNA and protein levels. Cell viability and proliferation ability were evaluated by CCK8 and soft agar colony formation assays. As shown in Figure 2E–2G, the ability of cell viability and colony formation were significantly greater in SPTBN1sh-transfected cells than in Con-sh cells and significantly lower in SPTBN1-V5-transfected cells than in Con-V5 cells.

The in vivo effects of SPTBN1 on the growth of EOC were explored using a xenograft tumor model. The tumor volumes and tumor weights were significantly increased in the tumors derived from LV-SPTBN1sh A2780 cells compared with LV-RFP A2780 cells (Figure 2H–2J).

Additionally, we found that knockdown of SPTBN1 expression inhibited the apoptosis of HO8910 cells (Supplementary Figure 1A, 1B). SPTBN1 suppression decreased the expression of the apoptosis inducer Bax and enhanced the expression of the apoptosis inhibitor Bcl-2 (Supplementary Figure 1B, 1C). To avoid the fluorescence of the vector, the cells were transfected with SPTBN1 siRNAs or control siRNA, detected by annexin V, and then analyzed by flow cytometry.

### SPTBN1 inhibits the migration of epithelial ovarian cancer cells

**Figure 3:**
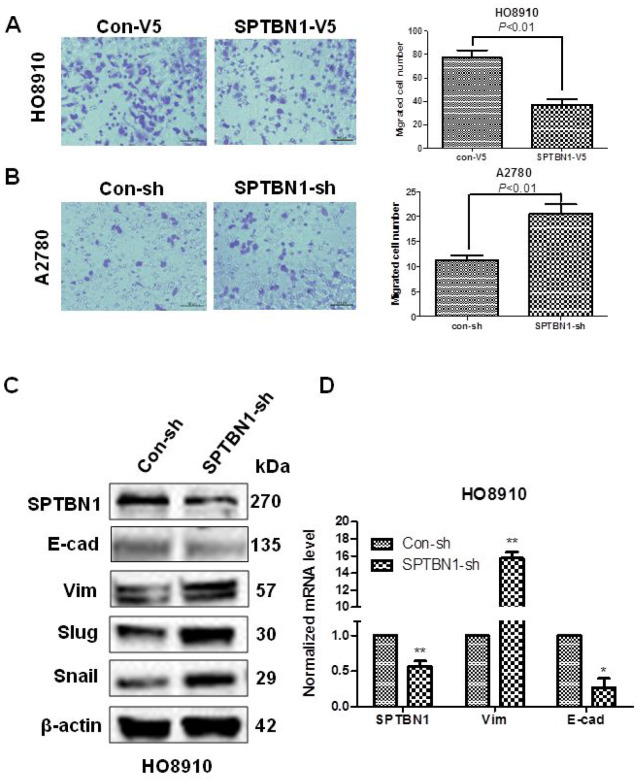
**SPTBN1 inhibits the migration and EMT of epithelial ovarian cancer cells.** (**A**, **B**) Assessments of cell migration. Overexpression of SPTBN1 inhibits the migration of HO8910 cells (**A**), while downregulation of SPTBN1 promotes the migration of A2780 cells (**B**). (**C**, **D**) Comparison of EMT-related markers at the protein level (**C**) and mRNA level (**D**). * *P* < 0.05, **P < 0.01 vs Con-sh, n=3.

The results showed that SPTBN1 overexpression inhibited the migration of HO8910 cells (Figure 3A), and SPTBN1 suppression promoted the migration of A2780 cells (Figure 3B). Suppression of SPTBN1 enhanced EMT in HO8910 cells by decreasing the EMT-related marker E-cadherin (E-cad) and increasing Vimentin (Vim), Slug and Snail expression at the protein (Figure 3C) and mRNA levels (Figure 3D).

### Positive correlation between the expression of SPTBN1 and SOCS3 in EOC patients and cell lines

**Figure 4:**
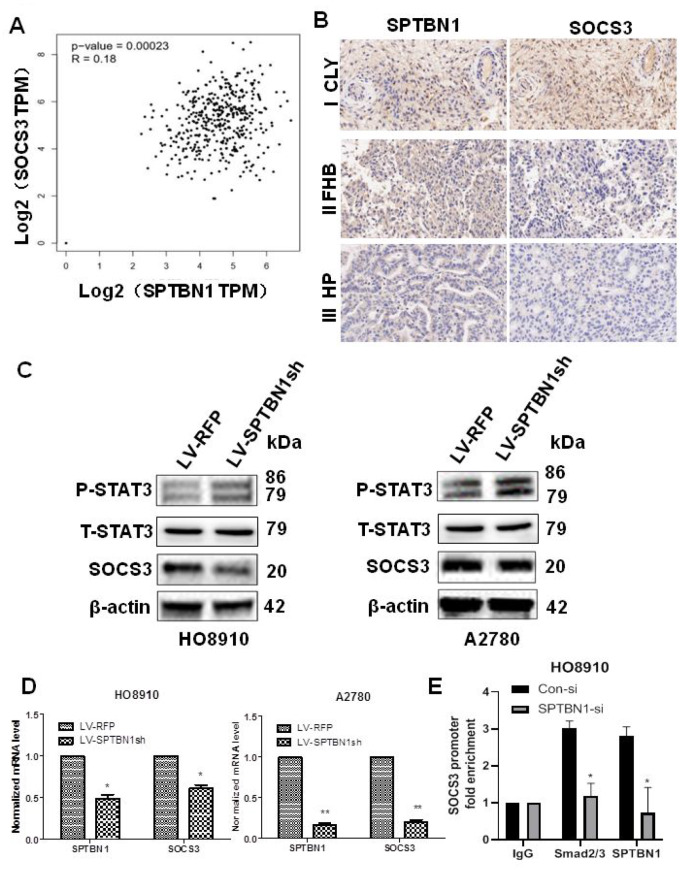
**Positive correlation between the expression of SPTBN1 and SOCS3 in epithelial ovarian cancer patients and cells. **(**A**, **B**) Positive correlation between SPTBN1 and SOCS3 expression was investigated by GEPIA (gene expression profiling interactive analysis, http://gepia.cancer-pku.cn) based on the TCGA dataset (**A**) and observed by IHC staining of ovarian cancer patient samples (n=11) with 40× magnification (**B**). (**C**) The protein level of SOCS3 was decreased, and the phosphorylated STAT3 level was increased when SPTBN1 was downregulated. (**D**) SPTBN1 regulated SOCS3 expression at the mRNA level. * *P* < 0.05, ** *P* < 0.01 vs LV-RFP. (**E**) ChIP-qPCR was performed in HO8910 cells with anti-SPTBN1 and anti-Smads2/3 antibodies to determine the enrichment of SOCS3 promoter region sequences in the obtained ChIP DNA. *P < 0.05 vs Con-si.

Database analysis (http://gepia.cancer-pku.cn/) and IHC staining of tumor tissue from EOC patients (n=11) showed that the expression of SPTBN1 was positively associated with SOCS3 in EOC (Figure 4A, 4B). Suppression of SPTBN1 downregulated SOCS3 expression in HO8910 and A2780 cells at the protein (Figure 4C) and mRNA levels (Figure 4D) and enhanced phosphorylation of STAT3, suggesting that the downregulated expression of SPTBN1 could activate the JAK/STAT signaling pathway in A2780 and HO8910 cells by reducing the expression of SOCS3. We further performed ChIP-PCR and demonstrated that downregulation of SPTBN1 in HO8910 cells impaired Smads2/3 binding to the promoter region of the SOCS3 gene (Figure 4E), which indicated that SPTBN1 can regulate SOCS3 at the transcriptional level through the TGF-β/Smads2/3 pathway.

### SPTBN1 inhibits the JAK/STAT signaling pathway through SOCS3

**Figure 5:**
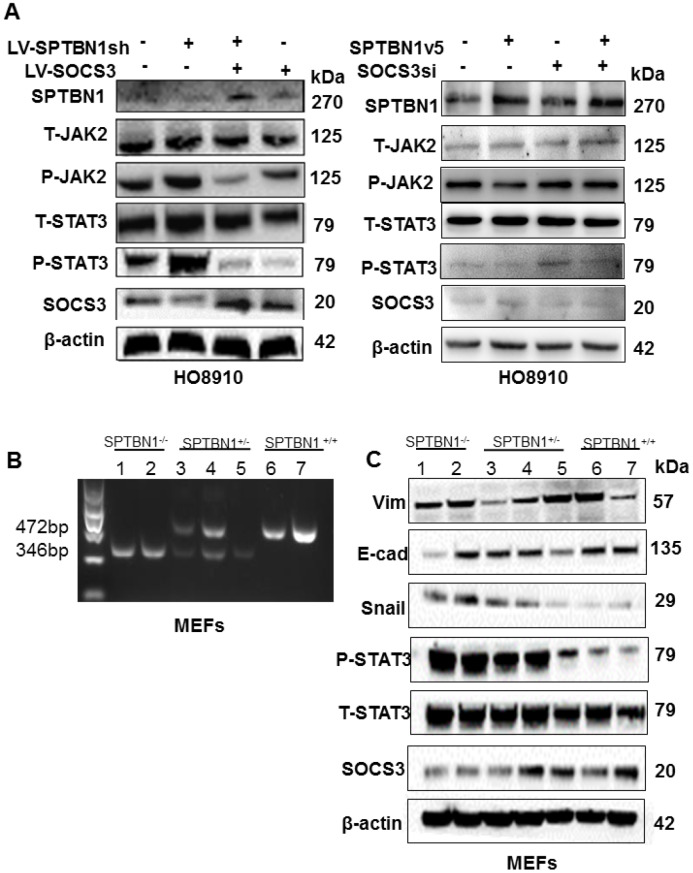
**Loss of SPTBN1 activates the JAK/STAT3 signaling pathway through downregulation of SOCS3.** (**A**) Assessments of JAK/STAT signaling pathway-associated proteins by western blot after SPTBN1 knockdown cooperated with SOCS3 overexpression in HO8910 cells (left), and SPTBN1 overexpression cooperated with SOCS3 knockdown (right). (**B**) DNA gel electrophoresis after PCR. Mouse embryonic fibroblasts (MEFs) were cultured from SPTBN1^-/-^ embryos (n=2), SPTBN1^+/-^ embryos (n=3) and wild-type embryos (n=2). The genotypes of MEFs were identified by PCR and DNA gel electrophoresis. Lanes 1 and 2: SPTBN1^-/-^ MEFs; lanes 3, 4, and 5: SPTBN1^+/-^ MEFs; lanes 6 and 7: SPTBN1^-/-^ MEFs. (**C**) Assessments of EMT and JAK/STAT3 signaling pathway-associated proteins by western blot in SPTBN1^-/-^, SPTBN1^+/-^, and SPTBN1^+/+^ MEFs. Lanes 1 and 2: SPTBN1^-/-^ MEFs; lanes 3, 4, and 5: SPTBN1^+/-^ MEFs; lanes 6 and 7: SPTBN1^-/-^ MEFs. Loss of SPTBN1 can promote EMT, inhibit SOCS3 and activate the JAK/STAT signaling pathway.

The results showed that overexpression of SOCS3 reversed the levels of p-JAK2 and p-STAT3, which were enhanced by SPTBN1 inhibition in HO8910 (Figure 5A, left) and A2780 cells (Supplementary Figure 2). Additionally, SPTBN1 overexpression resulted in decreased p-STAT3 and p-JAK2, while downregulation of SOCS3 in HO8910 cells reversed the effect of SPTBN1 overexpression on p-JAK2 and p-STAT3 (Figure 5A, right), validating the correlation between SPTBN1 and SOCS3. SPTBN1 overexpression further resulted in decreased p-STAT3 under the IL-6 activator at different time points (Supplementary Figure 3). It was then verified in mouse embryonic fibroblasts (MEFs) that SPTBN1 inhibited the JAK/STAT signaling pathway through SOCS3. MEF cells were cultured from SPTBN1^-/-^, SPTBN1^+/-^ and wild-type embryos, and their genotypes were identified by PCR and DNA agarose gel electrophoresis (Figure 5B). By western blot, we found that the expression of SOCS3 was decreased in SPTBN1^-/-^ MEFs, and the p-STAT3 level was elevated. Moreover, the expression of E-cadherin was decreased, and the expression of Vimentin and Snail was increased in SPTBN1^-/-^ MEFs (Figure 5C). These results demonstrated that SPTBN1 suppression could activate the JAK/STAT signaling pathway and promote EMT through inhibition of SOCS3 expression.

### The effects of SPTBN1 on EOC cell viability and migration are mediated by the SOCS3/JAK/STAT3 pathway

**Figure 6:**
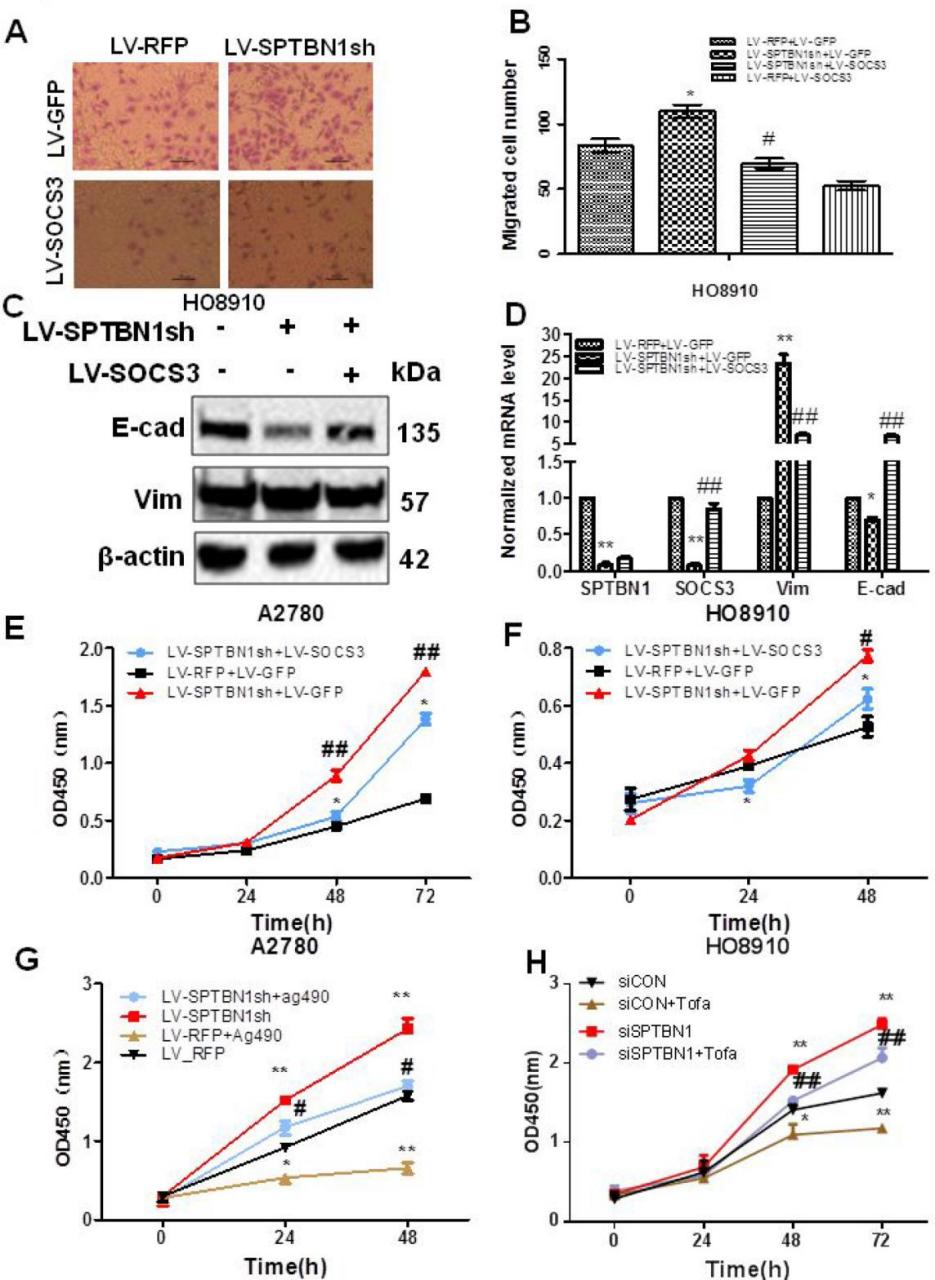
**SOCS3 overexpression or the JAK2 inhibitor reverses the inhibitory effects of SPTBN1 on cell viability and migration.** (**A**, **B**) In vitro cell migration assay. * *P* < 0.05 vs LV-RFP+LV-GFP, #*P* < 0.05 vs LV-SPTBN1sh+LV-GFP, n=3. (**C**, **D**) Comparison of protein (**C**) and mRNA (**D**) levels of the EMT-related proteins E-cadherin (E-cad) and Vimentin (Vim). The expression of SOCS3 and E-cadherin was decreased and Vimentin was increased by the loss of SPTBN1, while SOCS3 overexpression reversed the effects of the loss of SPTBN1. *P<0.05 **P<0.01 vs LV-RFP+LV-GFP, ##*P*<0.01 vs LV-SPTBN1sh+LV-GFP, n=3. (**E**–**H**) Cell viability was determined by CCK8 assay. SOCS3 overexpression reversed the enhanced cell viability due to the loss of SPTBN1 in A2780 (**E**) and HO8910 cells (**F**). #*P*<0.05, ##*P*<0.01 vs LV-RFP+LV-GFP, *P<0.05 vs LV-SPTBN1sh+LV-GFP, n=3. The JAK2 inhibitor Ag490 or tofacitinib (Tofa) inhibited cell viability and reversed the promoting effect of the loss of SPTBN1 in A2780 (**G**) and HO8910 cells (**H**). *P<0.05, **P<0.01 vs LV-RFP, #*P*<0.05 vs LV-SPTBN1sh, n = 3.

Next, we determined whether SOCS3 mediated the effects of SPTBN1 on the viability and migration of EOC cells. Our results showed that cell migration ability (Figure 6A, 6B) and viability (Figure 6E, 6F) were reversed after overexpression of SOCS3 in HO8910 and A2780 cells with SPTBN1 suppression. Mechanistically, exogenous expression of SOCS3 reversed the enhanced expression of Vimentin and decreased expression of E-cadherin induced by SPTBN1 inhibition in HO8910 cells at the mRNA and protein levels (Figure 6C, 6D). Similarly, the JAK2 inhibitor Ag490 (80 μg/ml) or tofacitinib (5 nM) inhibited the viability of A2780 or HO8910 cells and partially reversed the promoting effect of SPTBN1 suppression (Figure 6G, 6H). Our data indicated that SPTBN1 inhibited cell viability and migration in epithelial ovarian cancer by inhibiting the JAK/STAT signaling pathway through regulation of SOCS3.

## DISCUSSION

Epithelial ovarian cancer (EOC) is the leading cause of death among gynecologic cancers. EOC patients present with an advanced stage or even metastasis at initial diagnosis, and there is a high ratio of drug resistance and recurrence after conventional chemotherapy. It is critical to investigate genes involved in ovarian cancer metastasis to help identify new targets for ovarian cancer treatment.

As a cytoskeletal protein that maintains cell morphology and normal physiological functions, SPTBN1 is also involved in the malignant biological behavior of a variety of tumors, such as hepatocellular carcinoma, colorectal cancer, and pancreatic cancer [6]. SPTBN1 can regulate the cell cycle and EMT through TGF-β and Wnt/β-catenin signaling pathways, thus regulating the proliferation and migration of hepatocellular carcinoma. In addition, SPTBN1 can play an anticancer role by regulating apoptosis, DNA damage repair and angiogenesis [31]. In EOC, it was reported that SPTBN1 contributed to platinum resistance by forming a spectrin-GS-pt complex [18]. A proteomics study showed that a low level of SPTBN1 in EOC patients was associated with the loss of BRCA1 [19]. However, the effects and mechanisms of SPTBN1 on the progression and metastasis of epithelial ovarian cancer are still unknown.

In this study, we identified the relationship between SPTBN1 and the stage and prognosis of epithelial ovarian cancer by analyzing the TCGA database. The results showed that the expression of SPTBN1 gradually decreased with the progression of ovarian cancer, and the PFS of patients with low SPTBN1 expression was shorter than that in patients with high SPTBN1 expression. In addition, SPTBN1 expression was lower in more invasive HO8910-PM and ES2 cells than in HO8910 cells. The data indicate that SPTBN1 may promote the malignant biological behaviors of epithelial ovarian cancer. Using the EOC cell lines A2780 and HO8910, we verified that SPTBN1 inhibits the growth of epithelial ovarian cancer cells in vitro and in vivo and the migration of epithelial ovarian cancer cells in vitro.

The suppressors of cytokine signaling (SOCS) family has at least eight family members, which contain an SH2 domain and a SOCS box at the C-terminus. SOCS proteins are negative regulators of JAK/STAT signaling pathways through their interactions with phosphorylated JAKs and STATs to block signal transduction. In addition, SOCS can also reduce the stability of JAKs by promoting ubiquitination and proteasome degradation. Excessive activation of STATs can lead to an increase in SOCS gene expression due to a negative feedback mechanism. The JAK/STAT signaling pathway can regulate the expression of genes related to immunity, proliferation, differentiation, apoptosis and tumorigenesis [26, 32–37].

We have reported that the absence of SPTBN1 in hepatocellular carcinoma can activate Wnt pathways by downregulating kallistatin [6], while kallistatin increases apoptosis in breast cancer cells by inhibiting miR-203 and enhancing SOCS3 levels via PKC-ERK signaling [38]. In our study, we demonstrated that the expression of SPTBN1 and SOCS3 was positively correlated in EOC patients, and suppression of SPTBN1 activated the JAK2/STAT3 signaling pathway by downregulating SOCS3. Moreover, overexpression of SOCS3 or JAK2 inhibition reversed the promoting effects on cell viability and migration by downregulation of SPTBN1.

Chronic gynecological inflammation can damage the ovary and eventually induce ovarian cancer, and SOCS mediate cancer-associated inflammation [39]. SOCS3 can also function as an important regulator of genome stability by negatively regulating STAT3-dependent radioresistant DNA synthesis [40]. It should be investigated whether SPTBN1-regulated SOCS3 is involved in inflammation and radioresistance during ovarian cancer progression in future studies.

In conclusion, our study demonstrated that suppression of SPTBN1 promoted cell growth and migration in epithelial ovarian cancer by activating JAK/STAT signaling pathways, which are blocked by SOCS3. Thus, SPTBN1 may be a novel and useful candidate target for ovarian cancer treatment.

## MATERIALS AND METHODS

### Cell culture

The epithelial ovarian cancer cell lines A2780 and HO8910 were obtained from American Type Culture Collection (ATCC) and maintained in RPMI 1640 medium (Gibco, Carlsbad, CA, USA) supplemented with 10% fetal bovine serum (FBS, Gibco, Australia). Normoxic incubation (CO_2_ water-jacketed incubator; Thermo Electron, Waltham, MA) was performed at 37 °C in 5% CO2 with 95% humidity.

### Transfection and establishment of stable cell lines

A2780 and HO8910 cells were transiently transfected with SPTBN1-sh plasmid (Con-sh as the control) or with SPTBN1-V5 plasmid (Con-V5 as the corresponding control) using Lipofectamine 2000 transfection reagent according to the manufacturer's instructions (Invitrogen, Carlsbad, CA, USA). Forty-eight hours after transfection, the cells were used for subsequent experiments. SPTBN1 expression in A2780 cells and HO8910 cells was also stably downregulated by lentivirus vector LV-SPTBN1sh (LV-RFP as control) to conduct in vivo experiments. These vectors, the lentivirus expressing human SOCS3 (LV-SOCS3) and the control vector (LV-GFP) were purchased from GeneChem Co., Ltd (Shanghai, China).

### CCK8 assay

Cell proliferation was evaluated by CCK8 (Dojindo, Kumamoto, Japan) assay. A2780 cells were transfected with SPTBN1-sh or Con-sh, and HO8910 cells were transfected with SPTBN1-V5 or Con-V5 using Lipofectamine 2000 transfection reagent and cultured for 48 h. To determine if the effect of SPTBN1 is mediated by SOCS3, A2780 and HO8910 cells stably transfected with LV-SPTBN1sh (LV-RFP as control) were further transfected with LV-SOCS3 (LV-GFP as control) or pretreated with JAK2 inhibitor (Ag490 or tofacitinib) for 24 h. Cells were then seeded in 96-well plates (1×10^3^ cells/well) and cultured for the indicated time periods (0 h, 24 h, 48 h, 72 h) before addition of 10 μl CCK8 (5 mg/ml) reagent to each well. After 4 h of incubation at 37 °C, absorbance at 450 nm was measured with a Thermomax microplate reader (Molecular Devices, LLC, Sunnyvale, CA, USA).

### Soft agar colony formation

In brief, A2780 cells and HO8910 cells with stable suppression of SPTBN1 were harvested using trypsin and counted. Then, a soft agar colony formation assay was performed as previously described [41].

### Transwell cell migration assay

A2780 cells were transiently transfected with SPTBN1-sh or Con-sh, while HO8910 cells were transiently transfected with SPTBN1-V5 or Con-V5. Transwell cell migration assays were then performed as previously described [41]. To determine whether the effect of SPTBN1 is mediated by SOCS3, HO8910 cells stably transfected with LV-SPTBN1sh (LV-RFP as control) were then further transfected with LV-SOCS3 (LV-GFP as control) for 24 h. Cells were harvested using trypsin, counted and plated into the top chambers of Transwell plates (8 μm pore size; Corning, NY, USA).

### Western blot analysis

Western blot analysis was performed as previously described [41]. The primary antibody against SPTBN1 was from GenScript Corporation (Nanjing, China), and other primary antibodies against SOCS3, JAK2, p-JAK2 (Tyr1007/1008), STAT3, p-STAT3 (Tyr705) and EMT-related proteins were from Cell Signaling Technology (Danvers, MA, USA). β-Actin (Cell Signaling Technology, Danvers, MA, USA) as a loading control was also detected.

### Real-time quantitative PCR (qPCR)

Total RNA was extracted, and cDNA was synthesized using the ReverTra AceqPCR RT Kit (Toyobo, Osaka, Japan). The 10 μl qPCR system contained 4 μl (10 ng) cDNA, 1 μl (0.25 μM) forward and reverse primers, and 5 μl SYBR Green qPCR Master Mix (Bio-Rad, Hercules, CA, USA). The primers used were as follows: human SPTBN1, 5'-ATCTAACGCACACTACAACCTG-3' (forward), 5'-TCAAGCACCTTTCCAATTCGT-3' (reverse); SOCS3, 5'-CCAAGAACCTACGCATCCA-3' (forward), 5'-GGAGTCCAGGTGACCGTTG-3' (reverse); Bcl-2, 5'-TCCACCAAGAAGCTGAGCGAG-3' (forward), 5'-GTCCAGCCCATGATGGTTCT-3' (reverse); Bax, 5'-TTCTTTGAGTTCGGTGGGGTC-3' (forward), 5'-TGCATATTTGTTTGGGGCAGG-3' (reverse); Vimentin, 5'-CTCTCAAAGATGCCCAGGAG-3' (forward), 5'-GCACGATCCAACTCTTCCTC-3' (reverse); and E-cadherin, 5'-GGAGCAGAAAGCAGAACCC-3' (forward), 5'-TTCCTTCCACGAAACCAGTG-3' (reverse).

### Xenograft experiments

All studies involving experiments with animals were approved by the Ethics Committee of Experimental Research at Fudan University Shanghai Medical College, following the “Guide for the Care and Use of Laboratory Animals” published by the United States National Institutes of Health.

Six 6-week-old nude mice were injected subcutaneously in the bilateral flank area with 1×10^7^ A2780 cells stably transfected with LV-RFP or LV-SPTBN1sh (grouped as LV-Con or LV-SPTBN1sh) in 200 μl of phosphate-buffered saline (PBS). Tumor growth was monitored every 3 days to calculate tumor volume according to the formula V(mm^3^)=L×W^2^/2 (L is length and W is width). Three weeks after injection, the mice were euthanized with CO_2_, and the tumor weight was measured.

### Analysis of TCGA data for human ovarian cancer

The correlation between SPTBN1 expression and cancer stages was retrieved from UALCAN (http://ualcan.path.uab.edu/) by analysis of clinicopathological data available from The Cancer Genome Atlas (TCGA) project for human ovarian cancer. Progression-free survival (PFS, n=1435) and overall survival (OS, n=1656) were analyzed using TCGA data (probe 214856_at). The correlation between SPTBN1 expression and SOCS3 expression was assessed by Gene Expression Profiling Interactive Analysis (http://gepia.cancer-pku.cn/).

### Chromatin immunoprecipitation (ChIP) assay

The ChIP assay was carried out as described previously [41]. Chromatin fragments derived from HO8910 cells were immunoprecipitated with 5 μg of an antibody against SPTBN1 (GenScript Corporation, Nanjing, China) or an antibody against Smads2/3 (#8685T, Danvers, MA, USA). Real-time PCR analysis was performed with primers amplifying the promoter of SOCS3: 5'-ATCCCTGGCGTGCCTATTC-3' (forward) and 5'-TCGAGGTGGAACGATGGC-3' (reverse).

### Statistical analysis

The data are presented as the means±SEMs. Differences among groups were determined with one-way analysis of variance (ANOVA) with a post hoc test for multiple comparisons. Differences between two groups were assayed by two-tailed Student's t-test using GraphPad 5.0. Statistical significance was defined as P < 0.05.

## Supplementary Material

Supplementary Figures
