# Supplementary material for: SPTBN1 suppresses the progression of epithelial ovarian cancer via SOCS3-mediated blockade of the JAK/STAT3 signaling pathway
Source: Aging (Albany NY). 2020 Jun 8;12(11):10896–911. doi: 10.18632/aging.103303 (PMC7346039; doi:10.18632/aging.103303)
Supplement: Supplementary Figures [file aging-12-103303-s001..pdf]

SUPPLEMENTARY FIGURES

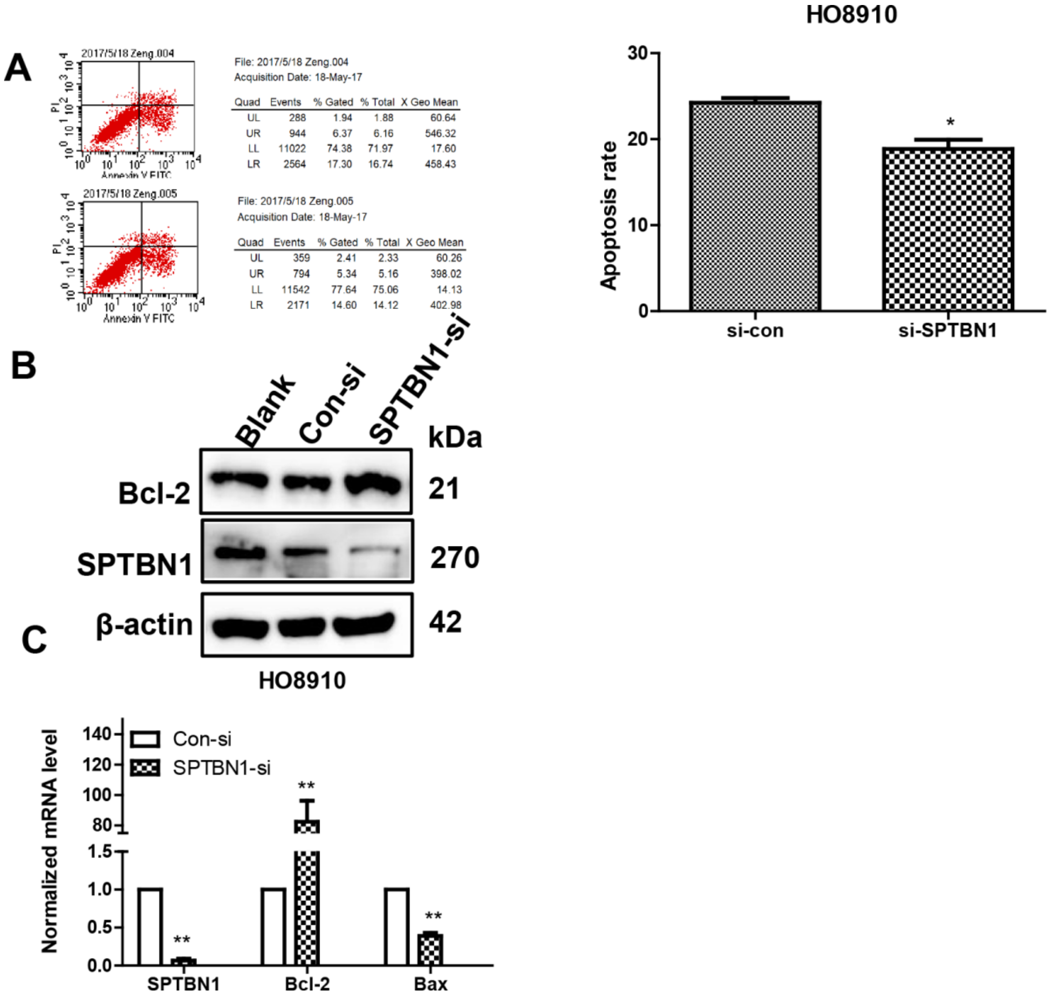

Supplementary Figure 1. (**A**) Apoptosis assay by FCM. SPTBN1 siRNA inhibited the apoptosis of HO8910 cells. (**B**, **C**) Expression of apoptosis-related genes was assessed by western blot and qPCR. In HO8910 cells without SPTBN1 expression, the expression of the apoptosis-promoting gene Bax was decreased, while Bcl-2, which inhibits apoptosis, was increased at the protein (**B**) and mRNA (**C**) levels. **P<0.01 vs Con-si, n=3.

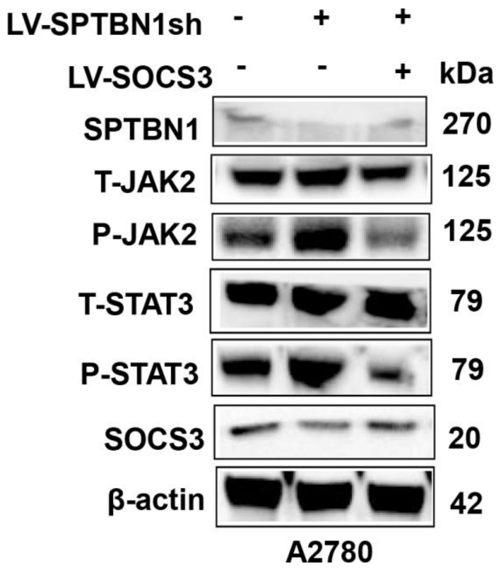

Supplementary Figure 2. Assessments of proteins associated with the JAK/STAT signaling pathway by western blot after SPTBN1 knockdown cooperated with SOCS3 overexpression in A2780 cells.

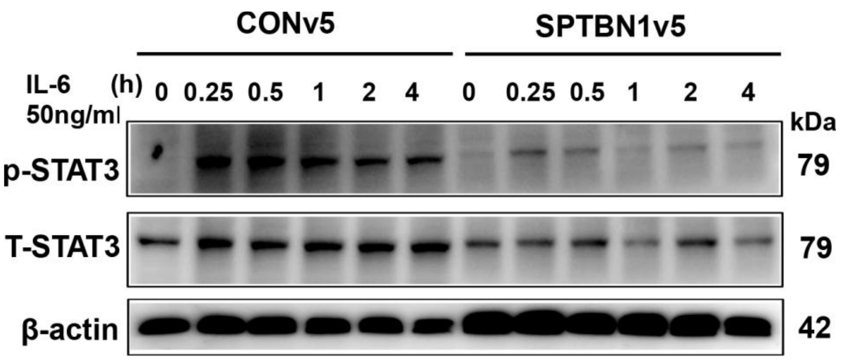

Supplementary Figure 3. IL-6, an activating cytokine, was used to determine STAT3 activation at different time points in HO8910 cells after SPTBN1 overexpression.
